# Management of adult attention deficit hyperactivity disorder in UK primary care: a survey of general practitioners

**DOI:** 10.1186/1477-7525-11-22

**Published:** 2013-02-22

**Authors:** Suzanne McCarthy, Lynda Wilton, Macey Murray, Paul Hodgkins, Philip Asherson, Ian CK Wong

**Affiliations:** 1 School of Pharmacy, University College Cork, Cork, Ireland; 2 Pharmacy Department, Cork University Hospital, Cork, Ireland; 3 Centre for Paediatric Pharmacy Research, School of Pharmacy, University College London, London, UK; 4 Shire Pharmaceuticals LLC, Wayne, Pennsylvania, USA; 5 MRC Social Genetic and Developmental Psychiatry, King’s College London, Institute of Psychiatry, London, UK; 6 Centre for Safe Medication Practice and Research, Department of Pharmacology and Pharmacy, Li Ka Shing Faculty of Medicine, The University of Hong Kong, Hong Kong, Hong Kong

**Keywords:** Attention deficit hyperactivity disorder, Adult, Stimulant, Primary care, General practitioner

## Abstract

**Background:**

Compared to existing literature on childhood attention deficit hyperactivity disorder (ADHD), little published adult data are available, particularly outside of the United States. Using General Practitioner (GP) questionnaires from the United Kingdom, this study aimed to examine a number of issues related to ADHD in adults, across three cohorts of patients, adults who received ADHD drug treatment in childhood/adolescence but stopped prior to adulthood; adults who received ADHD drug treatment in childhood/adolescence and continued treatment into adulthood and adults who started ADHD drug treatment in adulthood.

**Methods:**

Patients with a diagnosis of ADHD and prescribed methylphenidate, dexamfetamine or atomoxetine were identified using data from The Health Improvement Network (THIN). Dates when these drugs started and stopped were used to classify patients into the three cohorts. From each cohort, 50 patients were randomly selected and questionnaires were sent via THIN to their GPs.

GPs returned completed questionnaires to THIN who forwarded anonymised copies to the researchers. Datasets were analysed using descriptive statistics.

**Results:**

Overall response rate was 89% (133/150). GPs stated that in 19 cases, the patient did not meet the criteria of that group; the number of valid questionnaires returned was 114 (76%). The following broad trends were observed: 1) GPs were not aware of the reason for treatment cessation in 43% of cases, 2) patient choice was the most common reason for discontinuation (56%), 3) 7% of patients who stopped pharmacological treatment subsequently reported experiencing ADHD symptoms, 4) 58% of patients who started pharmacological treatment for ADHD in adulthood received pharmacological treatment for other mental health conditions prior to the ADHD being diagnosed.

**Conclusion:**

This study presents some key findings relating to ADHD; GPs were often not aware of the reason for patients stopping ADHD treatment in childhood or adolescence. Patient choice was identified as the most common reason for treatment cessation. For patients who started pharmacological treatment in adulthood, many patients received pharmacological treatment for comorbidities before a diagnosis of ADHD was made.

## Introduction

Attention deficit hyperactivity disorder (ADHD) is a neurodevelopmental disorder, which in the past was considered to be a condition restricted to childhood [1-3]. ADHD is now recognised as a valid diagnosis in adults as symptoms and impairments of ADHD are known to persist past childhood and adolescence [1,3-6]. ADHD continues to be diagnosed mainly in childhood, and for some patients, the condition will follow a remitting course as they get older and these patients may no longer experience the symptoms and impairments of ADHD in adulthood. For many whom no longer report symptoms, the decision to stop treatment is often taken by the adolescents themselves [7]. For others with ADHD diagnosed in childhood or adolescence, the symptoms and impairments persist into adulthood and they continue to seek medical treatment, which may include pharmacological treatment [7]. Some adults with ADHD are recognised, for the first time, to have ADHD-associated impairment in adulthood. In this situation, the diagnosis is based on the persistence of symptoms and impairments from childhood/adolescence into adult life [5].

Compared to younger patients, adults with ADHD are more likely to exhibit inattentive symptoms as hyperactive symptoms tend to diminish throughout the course of the condition [8]. However, they still suffer from symptoms such as the inability to sustain attention, disorganisation, forgetfulness and poor time management skills. In addition, a subset of adults with ADHD continue to have difficulties with overactive and impulsive symptoms; emotional dysregulation is increasingly being recognised as a related symptom that often co-occurs with untreated ADHD in adults [9]. There is now consensus amongst experts that ADHD, which is mainly diagnosed using the broader Diagnostic and Statistical Manual of Mental Disorders 4th Edition (DSM-IV) criteria, may currently affect at least 1-2% of adults in the United Kingdom (UK) and 2-4% worldwide [5,10-13]. The prevalence of ADHD in school-aged children and adolescents in the UK using DSM-IV criteria is estimated at 5% [14]. The prevalence of hyperkinetic disorder (HKD) in school-aged children and adolescents in the UK, defined by the more narrow International Classification of Disease 10th Revision (ICD-10) criteria is estimated at 1.5% [15].

While more evidence is being generated on the nature of ADHD in adulthood, ADHD guidelines published by the National Institute for Health and Clinical Excellence (NICE) in 2008 [3] state that ‘little empirical evidence is available to guide clinicians on questions such as the optimum duration of treatment, when it is appropriate to consider drug discontinuation and how and when to combine pharmacological and psychological treatments’. In addition, there are few data in the literature about the pharmacological management of ADHD in adults, particularly in the context of comorbid symptoms and syndromes, and particularly in Europe.

Through the use of a survey completed by general practitioners (GPs) about their patients, this study aimed to examine a number of issues concerning the diagnosis and treatment of ADHD in adults within the UK. For adults who received pharmacological treatment in childhood for ADHD and stopped this treatment before reaching adulthood, we sought to examine the reasons for stopping pharmacological treatment and to determine whether these patients experienced ADHD symptoms following cessation of treatment. Also whether they sought psychological interventions for ADHD and/or received pharmacological treatments for other mental health conditions following cessation of ADHD drug treatment.

For the group of patients who started treatment in childhood and continued to receive pharmacological treatment as an adult, our aim was to determine; whether these patients also received psychological treatment for ADHD, whether other mental health conditions were currently present or had ever been present and were treated as well as determining whether there was shared care between GP and specialist services. In the UK, shared care protocols outline ways in which the responsibilities for managing the prescribing of a medicine can be shared between the specialist and a primary care prescriber (GP) [14]. Shared care aims to facilitate seamless transition between primary and secondary care.

For those patients who started treatment for ADHD in adulthood, our aim was to examine whether they had, and were treated, for other mental health conditions both prior to and after the ADHD diagnosis was made, and also whether there were shared care arrangements between GPs and specialist services.

## Methods

### Data source

Data from The Health Improvement Network (THIN) database were used to identify patients that met the criteria for the study (described below). THIN contains anonymised computerised medical records entered by GPs in the UK for patient healthcare management. With coverage of approximately 5.7% of the UK population (2009), practices included in the database are broadly representative of practices in the UK in respect of patients’ demographics and characteristics [16]. GPs participating in THIN are trained to record medical records using the Vision general practice system (In Practice Systems; London, UK) and the validity of data on the database for research has been supported by a number of studies [17-20]. Those GPs who contribute to THIN and consent to provide further information on patients were included in the study. GPs received a nominal payment (£85) for each questionnaire completed and returned.

### Patient identification & selection

The method of patient selection has been described in detail elsewhere [21], in summary; patients who had a diagnosis of ADHD/HKD and a prescription record for methylphenidate, dexamfetamine or atomoxetine (approved drugs for the treatment of ADHD in the UK) coded on the THIN database during the study period 1st January 2003 to 31st December 2008 were identified. This sampling timeframe provided the most recent data available at the time the study was initiated (2009). The entire prescription records of the patients, which could include prescriptions for a study drug issued before 1st January 2003, were examined to determine at what age they received their first prescription for a study drug, whether they continued to receive prescriptions or had stopped, and if they had stopped, at what age they stopped treatment. As the purpose of the study was to examine issues relating to ADHD treatment in adulthood, of those patients identified above, only those who had reached the age of 18 years or greater during the study period were included. The age of patients was calculated as follows: once patients reach the age of 15 years, the date of birth is switched by THIN to the year of birth only (for patients younger than 15 years, the month of birth is also recorded). Therefore, as all patients included in the current study were aged 18 years or greater, the date of birth was imputed as 1st January and Year of Birth. Approximate ages of the patients were calculated at the mid-point of 2010 when questionnaire data were collected.

Identified patients (aged ≥ 18 years during the study period) were categorised into one of three cohorts:

• Adults who received pharmacological treatment for ADHD in childhood or adolescence but stopped before adulthood (Group 1)

• Adults who started pharmacological treatment for ADHD in childhood or adolescence and continued treatment into adulthood (Group 2)

• Adults who newly started pharmacological treatment for ADHD in adulthood (Group 3)

Patients in Group 1 consisted of adult patients who started ADHD treatment in childhood or adolescence (6–17 years) and stopped treatment before the age of 18 years. A total of 190 patients were identified. From this cohort, a random sample of 50 patients was selected.

Patients in Group 2 consisted of patients who started treatment in childhood or adolescence and were still receiving prescriptions at age ≥ 18 years. A total of 106 patients were identified. From this cohort, a random sample of 50 patients was identified.

Adult patients who started treatment at age 18 years or older were identified (n = 66 patients). Fifty patients were randomly selected to form Group 3.

**Figure 1:**
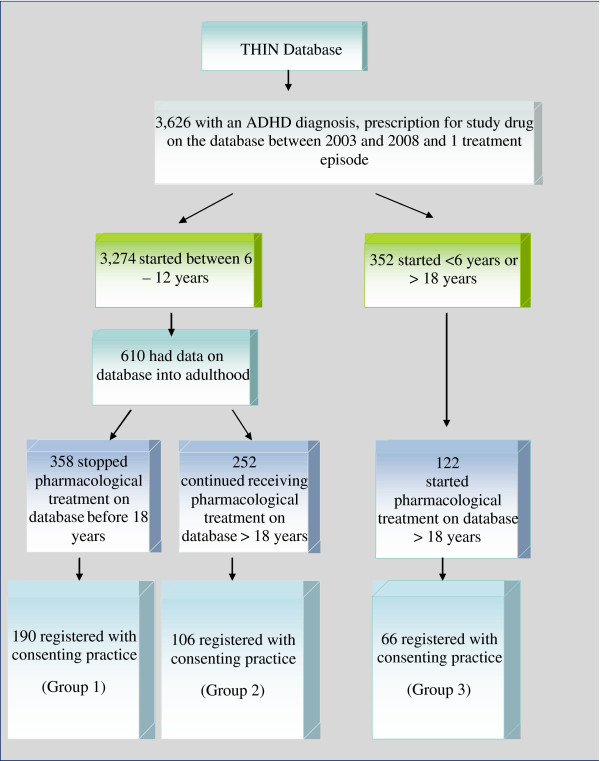
Flow chart to identify study participants.

A schematic of patient identification and selection is provided in Figure 1.

### Questionnaire design and distribution

The questionnaires were designed by the researchers (pharmacist (ICKW), pharmacoepidemiologist (MLM) and adult psychiatrist (PA)) to address the outcome measures below. Questionnaires were in a check box format, although respondents (GPs) had the opportunity to give additional information, using free text. For most questions, respondents could provide more than one answer. The questionnaires for the three cohorts were similar in terms of content and format e.g. patient demographics, however the context for some of the topic areas were specific to the patient cohort e.g. for patients who stopped ADHD treatment before adulthood (Group 1), questions regarding psychological therapy for ADHD refer to the period since stopping medication, whereas for patients in Group 2, those who continued treatment from childhood into adulthood, questions refer to patients receiving psychological therapy in addition to pharmacological therapy. GPs were provided with information on the symptoms of ADHD (as per NICE guidelines 2008) and information on psychotropic medications (as per British National Formulary). Copies of the questionnaires are available from the authors on request.

Researchers did not contact the GP practices directly. Three lists (50 cases for each group) with encrypted patient and practice ID codes were sent by the researchers to THIN who deciphered the practice codes and linked the selected, anonymised patient ID codes to their GP. THIN has no information which could identify a patient. THIN printed the questionnaires (with practice code and patient ID code) and sent them directly to the GP. The patient ID code enabled the GP to identify the individual patient(s). Each GP was requested to complete the questionnaire for their selected patient(s) and to send back all questionnaires to THIN. THIN ensured that any personal information (e.g. practice or hospital identities, anything which could reveal a doctor’s or patient’s location or personal information) was removed before forwarding a copy of the questionnaire to the researcher. Questionnaires forwarded to researchers only contained the encrypted patient ID (as originally sent to THIN) to link back to the original case selected; THIN retained the original questionnaires. The questionnaires were sent to a total of 90 GPs across the UK (71 – England; four – Northern Ireland; 11 – Scotland; four – Wales).

### Outcome measures

Group 1: Adult patients who started pharmacological treatment for ADHD in childhood or adolescence but stopped treatment before adulthood.

• The reasons for stopping medication.

• The percentage of adult patients who, since stopping ADHD medications, continued to report ADHD symptoms.

• The percentage of patients continuing to report ADHD symptoms, who then received psychological interventions for ADHD.

• The percentage of adult patients who, since stopping ADHD medications, receive/received pharmacological treatments for other mental health conditions.

• Whether GPs considered ADHD to be the primary mental health diagnosis.

Group 2: Adult patients who started pharmacological treatment for ADHD in childhood or adolescence and continued treatment into adulthood.

• The percentage of adult patients who receive/received psychological interventions for ADHD.

• The percentage of adult patients who receive/received pharmacological treatments for comorbidities and the identification of treated comorbidities.

• The extent to which shared care protocols exist between primary and secondary care.

• Whether GPs considered ADHD to be the primary mental health diagnosis.

Group 3: Adult patients who newly started pharmacological treatment for ADHD in adulthood

• The percentage of patients who received treatment for other mental health conditions before the diagnosis of ADHD was made.

• Identification of these mental health conditions and their treatment.

• The percentage of patients who receive/received treatment (pharmacological/psychological) for mental health conditions in addition to ADHD treatment.

• The extent to which shared care protocols exist between primary and secondary care.

• Whether GPs considered ADHD to be the primary mental health diagnosis.

### Questionnaire analysis

Data from completed GP questionnaires were collated and entered into two Microsoft Access® databases (first and second entry). The datasets were compared for data cleaning purposes and checked for internal consistency; any differences identified were checked against the source questionnaire. Only those questionnaires in which the GP confirmed that the patient met the group criteria (e.g. a patient in Group 1 had stopped ADHD pharmacological treatment prior to adulthood) were considered valid questionnaires for the study and were included in the analyses.

Descriptive analyses were performed on all valid questionnaires using Stata/SE version 11.0 (Stata Corp, College Station, Texas, United States). For each question in the questionnaires, the responses were analysed in respect of the total number of questionnaires returned for that group and in respect of the number of questionnaires where the data for that question were completed by the GP.

### Ethical approval

Ethical approval for this study was granted by the Cambridgeshire 4 Research Ethics Committee (ref: 09/H0305/81).

## Results

### Questionnaire response

Of the 150 questionnaires (50 per group) that were sent to 90 GPs, 133 (88.7%) were returned by 80 GPs (64 – England; three – Northern Ireland; nine – Scotland; four – Wales) by datalock; of these 115 (86.5%) were for male patients and 18 (13.5%) for female patients (Table 1).

**Table 1:** Questionnaire response and patient demographics

	**Group 1** – **stopped tx before adulthood**	**Group 2** – **continued tx from childhood into adulthood**	**Group 3** – **started tx in adulthood**
***Questionnaire Response***			
Number of Questionnaires Sent	50	50	50
Number of Questionnaires Returned	47	41	45
% Response Rate	94 (47/50)	82 (41/50)	90 (45/50)
Number of Returned Questionnaires in which GP confirmed that patients met criteria of the cohort (Valid Questionnaires)	44	37	33
Number of GPs sent questionnaires	40	38	43
Number of GPs who returned questionnaires	38	31	39
***Patient Demographics***	(n = 44)	(n = 37)	(n = 33)
Gender (male,%)	39 (88.6)	36 (97.3)	25 (75.8)
Mean Age (years) at Diagnosis for Males (SD)	9.4 (±3.0)	10.4 (±3.4)	28.7 (±9.6) 10.4
Mean Age (years) at Diagnosis for Females (SD)	12.0 (±1.4)	9*	31.0 (±5.7)
Mean Age (years) at time of data collection for Males (SD)	20.0 (±1.3)	20.6 (±1.2)	36.7 (±10.4)
Mean Age (years) at time of data collection for Females (SD)	20.5 (±1.1)	19.4*	38.9 (±11.2)
Diagnosis made by			
Psychiatrist (N)	30	25	27
Paediatrician (N)	11	7	0
Unknown by GP (N)	3	5	3
Other (N)	0	0	3

* No SD provided as data based on one patient.

Of these, 114 valid questionnaires were analysed for this study (44 in Group 1, 37 in Group 2 and 33 in Group 3) relating to 100 males (39 in Group 1, 36 in Group 2 and 25 in Group 3). The demographics of the patients are presented in Table 1.

### Group 1: patients who started pharmacological treatment for ADHD in childhood/adolescence and stopped before adulthood (n = 44)

i) Reasons for Stopping Medication

The reasons for stopping treatment were not known by GPs for 19 patients. Analyses were performed based on the 25 patients in whom the reason was reported by the GP. The main reasons cited for treatment cessation were patient choice (n = 14) and improvement in symptoms (n = 8). The reasons provided by GPs on why the patients stopped treatment are presented in Table 2.

ii) Percentage of patients who, since stopping ADHD medications, continued to report ADHD symptoms

**Table 2:** Reasons for stopping pharmacological treatment for ADHD before adulthood in patients from Group 1 (n = 44)

Reasons for stopping medication*	**Frequency**	**% Definite answer**^**¥**^
	**(n = 44)**	**[95% CIs]**
		**(n = 25)**
Patient did not want to continue medication (inc. stopping when finished exams)	14	56.0
		[34.9–75.6]
Patient had improvements in symptoms	8	32.0
		[14.9–53.5]
Patient experienced side-effects of medication	2	8.0
		[1.0–26.0]
Other – parental decision of ineffectiveness	1	4.0
		[0.1–20.4]
Unknown by GP	15	N/A
Unknown by GP – patient lost to follow-up	4	N/A

*Respondents could choose more than answer ^¥^ GPs provided a reason other than ‘Unknown’.

Two GPs answered ‘Unknown’ to this question and so analyses were performed on 42 patients. Three of the 42 patients (7.1%) experienced ADHD symptoms subsequent to stopping ADHD medications and for all three patients, their symptoms were reported to be of moderate severity. According to the GPs, the majority of patients (92.9%; 39/42) who stopped pharmacological treatment before adulthood did not report experiencing ADHD symptoms subsequently.

iii) Percentage of patients continuing to report ADHD symptoms, who then received psychological interventions for ADHD

Of the three patients who continued to report ADHD symptoms, one patient received psychological treatment.

iv) Percentage of patients who, since stopping ADHD medications, receive/received pharmacological treatments for other mental health conditions

Two GPs answered ‘Unknown’ to this question and so analyses were performed on 42 patients. Seven of the 42 patients (16.7%) received medication for other mental health conditions. Six of these patients were treated for mood disorder, one of whom was also treated for anger management. The seventh patient was treated for anxiety. All seven patients were treated with antidepressants. The majority of patients (83.3%; 35/42) did not receive pharmacological treatments for other mental health conditions, after stopping their ADHD medication.

v) ADHD as the primary mental health diagnosis

GPs were asked if they considered ADHD to be the primary mental health diagnosis for the patient. Over half of GPs (54.5%, 24/44) responded ‘Yes’ to this question. Eighteen GPs (40.9%) did not consider ADHD to be the primary diagnosis while two GPs did not know (4.6%).

### Group 2: adult patients who started pharmacological treatment for ADHD in childhood or adolescence and continued treatment into adulthood (n = 37)

i) Percentage of adult patients who receive/received psychological interventions for ADHD

This information was reported as ‘Unknown’ or not reported for five patients; therefore analyses were performed on 32 patients (Table 3). Eighteen patients (56.3%) had received/were receiving psychological interventions for ADHD. Only four (12.5%) adult patients were currently receiving psychological treatments, at the time of questionnaire completion.

ii) Percentage of adult patients who receive/received treatment for mental health comorbidities

**Table 3:** Number of patients in Group 2 who receive/had received psychological interventions for ADHD (n = 37)

**Receiving psychological intervention**	**Frequency**	**% Definite answer**^**¥**^
	**(n = 37)**	**[95% CIs]**
		**(n = 32)**
Yes – currently	4	12.5
		[3.5–29.0]
Yes – within the last year	2	6.3
		[0.8–20.8]
Yes – within the last 5 years	9	28.1
		[13.7–46.7]
Yes – more than 5 years ago	3	9.4
		[2.0–25.0]
No	14	43.7
		[26.4–62.3]
Unknown by GP/Information not completed	4/1	N/A

^¥^ GPs provided a reason other than ‘Unknown’.

GPs responded that this information was ‘Unknown’ for three patients. Of the remaining 34 patients, eight (23.5%) were stated to be receiving or had received treatment for comorbid conditions, although the majority (76.5%, 26/34) were not. Of these eight patients, three were currently receiving treatment; two had received treatment within the last five years and three patients received treatment more than five years previously. The conditions for which these eight patients were treated are presented in Table 4. Four of the eight patients had prescribed medications listed by the GP.

iii) Extent to which shared care protocols exist between primary and secondary care

**Table 4:** Patients in group 2 with comorbid conditions and treatments prescribed for these

**Comorbid conditions**	**Frequency (n = 8)**	**Treatment prescribed (n = 4)**
Obsessive Compulsive Disorder	2	Antidepressants (1)^¥^/ Unknown by GP (1) ^*^
Mood Disorder	1	Antidepressants (1) ^¥^
Personality Disorder	1	Methylphenidate (1) ^Δ^
Learning Difficulties	1	No medication ^¥^
Aspergers	1	Unknown by GP ^*^
Self-harm	1	Antipsychotics (1) ^Δ^
Other – intracranial bleed, facial palsy, hemiplegia	1	Unknown by GP ^*^

^¥^- receiving treatment currently, ^Δ^ - within last 5 years, ^*^ - more than 5 years ago.

For fewer than 20% of the patients (18.9%, 7/37) the pharmacological treatment for ADHD was provided under a shared care protocol with another healthcare professional.

iv) ADHD as the primary mental health diagnosis

GPs were asked if they considered ADHD to be the primary mental health diagnosis for the patient. Over three-quarters of GPs (78.4%, 29/37) responded ‘Yes’ to this question. Six GPs (16.2%) did not consider ADHD to be the primary diagnosis while two GPs did not know (5.4%).

### Group 3: patients who newly started pharmacological treatment for ADHD in adulthood (n = 33)

i) Percentage of patients who received treatment for other mental health conditions before the diagnosis of ADHD was made

This information was reported as ‘Unknown’ by GPs for two patients and so analyses were based on the remaining 31 patients. Twenty adult patients (64.5%) had been treated for other mental health conditions before they were diagnosed with ADHD (see below). The remaining 11 patients (35.5%) had not received treatment for other mental health conditions.

ii) Identified mental health conditions and their treatment (pharmacological/psychological)

GPs were asked to list other mental health conditions diagnosed and treated. Twelve of these 20 patients were treated for a single condition, six patients for two conditions, one for three conditions and one for four conditions. The three most frequently reported conditions for which patients were treated were; mood disorder (13), personality disorder (five) and anxiety disorder (four). The treated mental health conditions and the modality of treatment are listed in Table 5. Of the 20 patients, 18 received pharmacological treatment (90%) of whom eight also received psychological treatments. The other two patients received psychological treatment only (10%).

iii) Percentage of patients who receive/received treatment (pharmacological/psychological) for mental health conditions in addition to ADHD treatment

**Table 5:** Patients in group 3 with mental health conditions prior to and in addition to an ADHD diagnosis

	**Patients in group 3 with mental health conditions *prior to being diagnosed with ADHD *(n = 20)**						**Patients in group 3 who were treated for mental health conditions *in addition to ADHD treatment *(n = 17)**	
**Patient**	**Mental health conditions**				Medications prescribed*	**Psychological Interventions**	Medications prescribed*	**Psychological interventions**
**1**	Mood Disorder	Personality Disorder	Substance abuse	Depression	Antidepressants, Antipsychotics	Yes	Antidepressants, Sedatives, Anxiolytics as required	Yes
**2**	Anxiety Disorder	Personality Disorder	Bulimia Nervosa		Antidepressants	Yes	No	No
**3**	Anxiety Disorder	Personality Disorder			No	Yes	No	No
**4**	Mood Disorder	Anxiety Disorder			Antidepressants	Yes	No	No
**5**	Mood Disorder	Substance Abuse			Antidepressants, Sedatives	Unknown by GP	Antidepressants	Yes
**6**	Mood Disorder	Personality Disorder			Antidepressants	Yes	No	Yes
**7**	Down’s Syndrome	Behavioural Problems			Sedatives	No	No	No
**8**	Personality Disorder	Substance Abuse			Antipsychotics, Sedatives	No	Antipsychotics, Sedatives, Other-trihexyphenidyl, Pregabalin	Unknown by GP
**9**	Anxiety Disorder				Antidepressants, Mood Stabilizers, Antipsychotics, Sedatives	Yes	Antidepressants, Antipsychotics, Sedatives	Yes
**10**	Bipolar Disorder				Antidepressants	Yes	Antipsychotics, Sedatives	Yes
**11**	Mood Disorder				Antidepressants, Antipsychotics	No	Antipsychotics	Unknown by GP
**12**	Mood Disorder				Antidepressants, Antipsychotics	No	Antidepressants	No
**13**	Mood Disorder				Antidepressants	No	Antidepressants	No
**14**	Mood Disorder				Antidepressants	No	No	No
**15**	Mood Disorder				Antidepressants	Yes	No	Yes
**16**	Mood Disorder				Antidepressants	Yes	Antidepressants	Yes
**17**	Mood Disorder				Antidepressants	No	Antidepressants	Yes
**18**	Mood Disorder				Mood Stabilizers	No	No	No
**19**	Mood Disorder				No	Yes	Antipsychotics	Yes
**20**	Learning disabilities				Antidepressants, Sedatives, Melatonin	No	Antidepressants, Mood Stabilizers, Antipsychotics	No
**21**^**¥**^	No					No	Antidepressants	Yes
**22**^**¥**^	No					No	Antidepressants	Unknown by GP
**23**^**¥**^	No					No	No	Yes

*It was not specified whether these were prescribed concurrently or sequentially.

^¥^ - patients not treated for other mental health conditions prior to ADHD diagnosis.

Seventeen adult patients (51.5%) were receiving or had received treatment for other mental health conditions in addition to ADHD treatment. Fourteen patients received pharmacological treatment, eight of whom also received psychological treatment. Three patients received psychological treatment only. Sixteen patients (48.5%) had not received treatment for mental health conditions in addition to ADHD treatment.

Of the 18 patients who were prescribed pharmacological treatment for other mental health conditions before the diagnosis of ADHD was made, 11 continued to be prescribed pharmacological treatment for other mental health conditions in addition to pharmacological treatment for ADHD.

iv) Extent to which shared care protocols exist between primary and secondary care.

For 39% of the patients (13/33) the pharmacological treatment for ADHD was provided under a shared care protocol with another healthcare professional.

v) ADHD as the primary mental health diagnosis

GPs were asked if they considered ADHD to be the primary mental health diagnosis for the patient. Over 40% of GPs (42.4%, 14/33) responded ‘Yes’ to this question. Ten GPs (30.3%) did not consider ADHD to be the primary diagnosis while nine GPs did not know (27.3%).

## Discussion

Using data from a computerised UK primary care database, of the 610 adult patients identified who had started ADHD drug treatment in childhood, 358 (58.7%) of these patients had stopped treatment before the age of 18 years, 252 (41.3%) continued ADHD drug treatment from childhood into adulthood. Also 122 adult patients who had started ADHD drug treatment in adulthood were identified. Data from questionnaires sent to the GPs of a random sample of 50 patients from each of these three distinct patient groups, were used to address issues with ADHD drug treatment from childhood into adulthood (Groups 1 and 2) and in adulthood (Group 3).

A high response rate was achieved in this study ranging from 82% to 94% across the three study groups. There are a number of factors which are likely to contribute to the high response rate: the GPs who contribute data to the THIN database, and who consent to providing additional patient information, are a self-selected group and in addition, those who completed and returned the questionnaire were compensated. A high response rate (>95%) using the THIN database has been reported elsewhere [18,22]. These response rates are higher than reported in other GP studies; a review of published GP studies from the early 1990s reported a mean response rate of 61% [23]. These authors acknowledged that this figure was likely to overestimate the true response rate as many studies with low response rates fail to get published.

### Reasons for stopping ADHD pharmacological treatment before adulthood (Group 1)

In the current study, GPs were not aware of the reason for stopping treatment in over one-third of patients. GPs, who were aware of the reason(s), stated that it was the patient’s decision to stop treatment in 56% of cases (14/25 who responded to this question). A qualitative study conducted by Wong et al. [7] in which patients were asked directly for reasons for cessation of treatment in adolescence reported in seven out of ten cases, the decision to stop treatment was their own and was often influenced by more than one factor. In addition, some patients who stopped treatment did so because they felt they were able to control themselves better than they could when they were younger, whilst others had gravitated towards environments and employment that did not place the same demands on maintaining focus and concentration.

While it is recognized that symptoms of ADHD decline over time in some patients, the prognosis of ADHD depends on the definition of persistence used [4,24]. Studies which have examined the persistence of the condition, using the symptomatic definition of remission, reported that the persistence of ADHD into adulthood was approximately 65% [4]. This current study was not designed to estimate persistence of ADHD into adulthood, but according to the patients’ GP, three of the 42 patients who stopped treatment before adulthood reported experiencing ADHD symptoms after stopping pharmacological treatment. Hence it may be prudent for GPs to review, on a regular basis, older adolescents who stop medication for ADHD in order to identify if symptoms and impairments return and thus whether these patients require further assessment and/or re-initiation of treatment.

Another reason cited for stopping treatment was the occurrence of side-effects, albeit in a low number of patients (2/25). The three regulatory approved drugs in the UK to treat ADHD have recognised and well established side-effect profiles including (but not limited to) effects on sleep, decreased appetite and the cardiovascular system. Many of the common side-effects of these drugs are transient and subside with time or with reduced dosage, however serious/severe events may warrant discontinuation of treatment. Although there have been some concerns of the safety of ADHD treatment, it is generally accepted that serious adverse reactions are uncommon [25,26]. In the present study, details of the nature and severity of the side-effects which resulted in discontinuation of the drug were not reported by the GPs.

Other reasons contributing to patient cessation of treatment which have been cited in the literature include the poor provision of treatment services for older adolescents and young adults. Typically in the UK, patients with ADHD are under the care of child and adolescent mental health services up to the age of 16 years or school-leaving age with GPs responsible for prescribing ADHD medications [27]. However, ADHD services within the adult mental health system in the UK are currently poorly developed [28] and clear arrangements for transition are often lacking [29]. Wong et al. reported this as a reason for stopping treatment in three out of ten patients interviewed [7].

### Psychological treatment for ADHD (Groups 1 & 2)

There are a number of psychological treatments available for patients with ADHD, as an alternative to or in combination with pharmacological treatment. The NICE guidelines acknowledge that there is scant information available on the use of this modality of treatment [3]. The latest meta-analysis on nonpharmacological interventions for ADHD indicate that better evidence for efficacy from blinded assessments is required for behavioral interventions, neurofeedback and cognitive training before they can be supported as treatments for core ADHD [30]. Psychological treatments for ADHD are currently likely to be limited to certain areas of the UK where specialist services are available to provide such interventions and this is especially true for adult patients for whom there are few specialist ADHD clinics [3].

In Group 1, GPs responded that 33% of patients received psychological interventions for ADHD following cessation of treatment. In Group 2, 56.3% of patients had ever received psychological therapy in addition to pharmacological treatment for ADHD; 12.5% of patients were currently receiving therapy.

Data from the current study showed great variability between these two groups; however this may be in part due to the small sample size of the individual groups. This variability suggests that psychological treatment may be considered either less favoured or less widely available for the treatment of ADHD.

### Treatment of other mental health conditions (Groups 1, 2 & 3)

The treatment of other psychological/psychiatric conditions was investigated in the current study, with results varying across the three patient groups.

The results demonstrate that in this sample of patients, adult patients who initiated treatment for ADHD in adulthood were more frequently treated for other mental health conditions prior to diagnosis of ADHD compared to patients who started pharmacological treatment for ADHD in childhood. The NICE report states that “In adults, coexisting symptoms, syndromes and disorders are frequently found to exist alongside the core ADHD syndrome, but their distinction from ADHD and the reasons for high rates of coexistence are not well addressed in the current literature” [3]. A recent review paper by Asherson and colleagues highlight the many difficulties associated with the presentation of ADHD in adults, with a particular emphasis on high-functioning individuals, the challenges of current diagnostic criteria, cultural influences on the recognition of the disorder, and the burden of ADHD, both socially and economically, on adults with the disorder [31]. An interview study, conducted by Matheson and colleagues, examining adult ADHD patients’ experiences of impairment, accessing services and treatment management, reported on the psychosocial burden from a delayed diagnosis of ADHD in adulthood [32]. Patients had sought help from services in childhood/adolescence due to depression or anxiety, which resulted in years of misdiagnoses and ineffective treatments; many patients attributed the cause of comorbid disorders to underlying ADHD. The effect of the delayed diagnosis was a chronic sense of failure and missed potential in many areas of their lives [32].

### Shared-care arrangements (Groups 2 & 3)

NICE guidelines, published in 2008, recommend that “drug treatment should only be initiated by an appropriately qualified healthcare professional with expertise in ADHD and should be based on a comprehensive assessment and diagnosis. Continued prescribing and monitoring of drug treatment may be performed by general practitioners, under shared care arrangements” [3]. However, although the questionnaire for the current study was administered in 2010, the data obtained related to the study period 2003 to 2008, therefore for the majority of patients, the NICE guidelines were not in place when these patients were receiving treatment. The data from the study revealed that at that time, shared-care was not in place for all patients receiving treatment for ADHD in adulthood (Groups 2 & 3). For those patients in Group 2, who started treatment in childhood/adolescence, fewer than 20% were treated under shared care protocols; however of those patients who started in adulthood (Group 3) approximately 40% of patients had shared care. It was not possible to examine any further differences e.g. geographical differences in the provision of shared care across the UK; however it is recognised that ADHD services within adult mental health care are currently poorly developed in the UK [28]. NICE also emphasized the benefit that such a service would bring including ‘better care of young people with ADHD during the transition between child and adolescent mental health services (CAMHS) or paediatric services, and adult mental health services’, ‘decreasing the number of people who disengage from services’, and ‘improving the recognition, diagnosis and treatment of ADHD in adults and reducing the risk of misdiagnosis’ [33]. It would be important in future studies to examine the provision of shared care and the impact this has on the delivery of care to patients with ADHD.

### Strengths and limitations

This study utilised THIN data to access information on a large cohort of patients with ADHD, across a wide age range, who were treated in primary care. The services provided by THIN also permitted the distribution and retrieval of anonymised questionnaires from GPs which enabled the analysis of information that is not routinely coded in automated databases. The response rate achieved was high, over 80% in all three groups and over 90% in two of the groups. A limitation of the study was the lower proportion of patients in Group 3 (adults who started ADHD treatment in adulthood) who met the study criteria; 73% in this group, compared with 94% in Group 1 and 90% in Group 2. Patients in the latter two groups were identified as having received their first prescription for a study drug in childhood or adolescence and followed forward into adulthood. Patients in Group 3 were also identified from the coded records on the database. However, in responding to the questionnaire, their GPs may have accessed information not coded on the database which indicated an earlier (pre-adulthood) prescription(s) for the treatment of ADHD. Historical data on childhood disorders and their treatment may be missing if patients move practices as adults; this may account for the difference in the number of valid cases between coded information and the response to the questionnaires, and highlights the importance of verifying electronic records with GP questionnaires.

For a number of questions asked, the answers were not known by the GP. This was particularly the case when GPs were asked for the reasons why patients in Group 1 stopped ADHD medication. It should be considered that there may be systematic differences between those patients for whom the reason was known such as engagement with GP, etc. In addition to the small sample sizes in each group, these missing data may be a source of bias to the study. However, the fact that GPs don’t know the reason for cessation of treatment in many cases is important information and reflects management practices for adult ADHD that could be improved upon.

While this study included three cohorts of patients, namely those who stopped ADHD treatment before adulthood, those who started treatment in childhood and continued treatment into adulthood and those who started treatment in adulthood, we were not able to investigate the long-term outcomes of these groups due to the time period of this study. It requires long-term monitoring to examine issues such as the benefits or risks of extended use of pharmacological treatment on symptoms, impairments, and quality of life for adult patients with ADHD.

Finally, although a large database was used to identify patients, the small sample sizes in each group warrants caution if data were extrapolated to the greater UK population.

## Conclusions

A number of points for consideration were identified from this study. The most frequent reason provided for stopping treatment was patient choice not to continue medication, followed by improvement in symptoms, in the cohort of adults who received pharmacological treatment in childhood and stopped this treatment before reaching adulthood. A small number of patients reported that they experienced symptoms of ADHD of moderate severity following cessation of pharmacological treatment; however only one patient received psychological therapy for the treatment of these symptoms. The majority of patients did not receive pharmacological treatments for comorbid conditions after stopping ADHD medications.

Psychological treatments for ADHD were used by 12.5% of patients who started treatment in childhood and continued to receive pharmacological treatment in adulthood, at the time of questionnaire completion. Fewer than one-quarter of these patients received pharmacological treatment for comorbid mental health conditions, while fewer than 20% of adults were receiving care under shared care arrangements.

Almost two-thirds of patients were treated for another mental health condition before the diagnosis of ADHD was made in adulthood; the majority of whom received pharmacological treatment. Approximately half of patients received treatment for a mental health condition in addition to receiving ADHD treatment. Fewer than half of the patients were cared for under shared care arrangements between primary care and specialist adult services.

This study has highlighted a number of key areas in the treatment of ADHD patients, including the diagnosis of ADHD in adults, the treatment of comorbid mental health conditions and the delivery of care through shared-care arrangements that warrant further research.

## Competing interests

LW and MM declare that they have no competing interests. SM received research funding as a result of involvement in this research study. PH is an employee and stock holder of Shire Pharmaceuticals LLC. Shire Pharmaceuticals develops and manufactures drugs to treat psychiatric conditions, including ADHD. PA has acted in an advisory role for Shire, Janssen-Cilag, Eli Lilly and Flynn Pharma. He has received education or research grants from Shire, Janssen-Cilag and Eli-Lilly. He has given talks at educational events sponsored by the above companies. ICKW was a member of National Institute for Health and Clinical Excellence (NICE) ADHD Guideline Group. ICKW has received funding from various pharmaceutical companies; however, none of this funding is related to this study.
